# The Current Situation and Development Prospect of Whole-Genome Screening

**DOI:** 10.3390/ijms25010658

**Published:** 2024-01-04

**Authors:** Caiting Yang, Yu Lei, Tinglin Ren, Mingze Yao

**Affiliations:** Shanxi Provincial Key Laboratory for Medical Molecular Cell Biology, Key Laboratory of Chemical Biology and Molecular Engineering of Ministry of Education and Institute of Biomedical Sciences, Shanxi University, Taiyuan 030006, China; yangcaiting@sxu.edu.cn (C.Y.); leiyu17835704018@163.com (Y.L.); tinglinr@163.com (T.R.)

**Keywords:** genome screening, CRISPR screen, RNAi, cDNA

## Abstract

High-throughput genetic screening is useful for discovering critical genes or gene sequences that trigger specific cell functions and/or phenotypes. Loss-of-function genetic screening is mainly achieved through RNA interference (RNAi), CRISPR knock-out (CRISPRko), and CRISPR interference (CRISPRi) technologies. Gain-of-function genetic screening mainly depends on the overexpression of a cDNA library and CRISPR activation (CRISPRa). Base editing can perform both gain- and loss-of-function genetic screening. This review discusses genetic screening techniques based on Cas9 nuclease, including Cas9-mediated genome knock-out and dCas9-based gene activation and interference. We compare these methods with previous genetic screening techniques based on RNAi and cDNA library overexpression and propose future prospects and applications for CRISPR screening.

## 1. Introduction

Genetic screening technologies are undoubtedly transforming basic as well as clinical and biotechnological research. Researchers routinely apply high-throughput screening techniques to identify genes that influence a specific phenotype in an unbiased fashion. The application areas of genetic screening technology extend beyond large-scale functional screenings, such as therapeutic applications, characterizing functional distal enhancers, targeted reprogramming of lineage specification, generation of induced pluripotent stem cells, and reversal of HIV latency [1,2,3,4,5,6,7,8]. In addition to traditional screening techniques, including RNAi and the overexpression of cDNA/ORF, the simple and efficient gene-targeting capacity of clustered regularly interspersed short palindromic Repeats (CRISPR) has been harnessed to functionally screen a large number of genes at the same time. Due to its robustness and flexibility, CRISPR is becoming a powerful high-throughput assay with applications that are transforming not only genome-editing studies, but also identifying the causal link between epigenetic marks and gene expression. The history of molecular biology has placed CRISPR-Cas9 among the major tools that enabled breakthrough discoveries and methodological advancements in science.

This review introduces traditional screening technologies and CRISPR screen, including CRISPRa, CRISPRko, CRISPRi, and base editing screening technologies. We discuss the developments and problems of screening delivery systems and bioinformatics analysis methods. However, for the most part, the review focuses on the CRISPR technology itself. Moreover, we review several key developments of the joint applications of genetic screen with other technologies and discuss the existing problems and development direction. Finally, we discuss the future prospects and the required technological advances.

## 2. Traditional Screening Techniques

### 2.1. RNAi Screening

RNA interference (RNAi) is an effective method that is widely used in functional genomics [9,10,11], including genetic screening, genetic function identification, signal transduction, and gene therapy. RNAi refers to the efficient and specific degradation of mRNA induced by double-stranded RNA (dsRNA) of identical sequences, finally leading to gene silencing. RNAi is often used together with high-throughput sequencing. At present, three types of RNA molecules have been identified: (1) short interacting RNA (siRNA), (2) short hairpin RNA (shRNA), and (3) microRNA (miRNA). These processes act on mRNA, causing mRNA degradation or the inhibition of mRNA translation.

**Figure 1:**
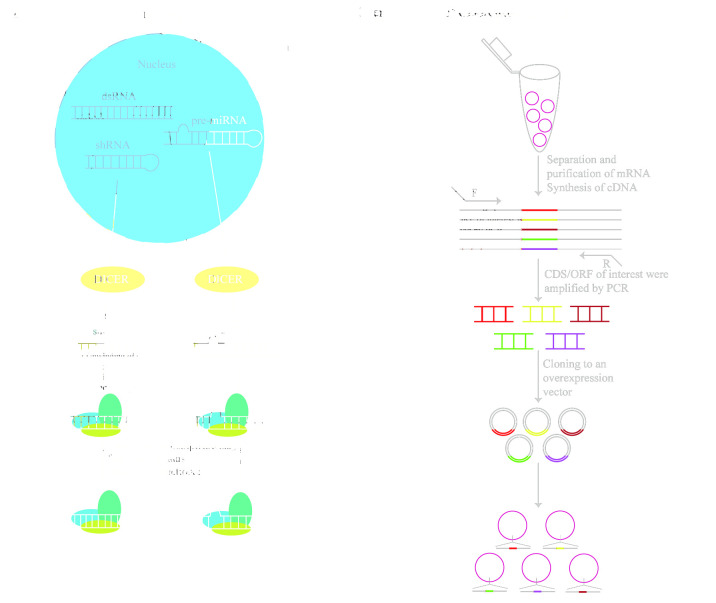
The basic working principle of traditional genetic screening techniques. (**A**) RNA interference (RNAi): cells transfected with double-stranded RNA (dsRNA) and small hairpin RNA (shRNA) are treated with the endoribonuclease Dicer complex enzyme to form short interfering (siRNA), which then binds to the RNA-induced silencing complex (RISC). The functional chain of the siRNA guides RISC to the mRNA target. The RISC complex prevents the synthesis of specific proteins via the degradation of the mRNA. Pre-miRNA processed with Dicer enzyme can be incorporated into the leaving RNA strand on RISC. miRNA-RISC then pairs with the target mRNA in an incomplete complementary manner, finally leading to mRNA degradation or the inhibition of mRNA translation. (**B**) Overexpression of complementary DNA/open reading frames (cDNA/ORFs): the coding sequence/open reading frame (CDS/ORF) of the target gene (different colors), once obtained, can be cloned into the overexpression vector, which is then introduced into the cell. Finally, a randomly or site-directed insertion into the host cell genome is carried out to achieve the specific overexpression of genes.

At present, the RNAi screening process consists of the following steps: (1) intracellular delivery of RNA molecules, (2) selection of transduced cells based on drug resistance or fluorescence, (3) screen enriching or sharply reducing RNA molecules to determine functional target genes according to the phenotypes (drug resistance, cell proliferative potential, survival ability, marker genes, among others). Screening based on siRNA is generally used for arrayed screening, in which siRNA is mixed with transfection reagents that are added to a cell suspension for transfection (Figure 1A). Because of the different characteristics of RNA molecules (Table 1 and Table 2), shRNA is mostly screened by pooling, and siRNA is usually screened by array.

RNAi technology can specifically interfere with the expression of target genes. It can be flexibly combined to inhibit multiple target genes concurrently and can be used for large-scale genetic screening. RNAi is reversible since the target genes are only temporarily knocked down at the mRNA level. Moreover, RNAi can knock down genes relying on the endogenous mechanism of cells with no need for Cas9 nuclease. Therefore, RNAi is suitable for cells with low efficiency in delivering Cas9/dCas9 [12]. In recent years, RNAi screening has become an important means of genetic screening [13,14]. Recently, Kristijonas et al. used a small hairpin (shRNA) to screen modifier regulators of hematopoietic stem and progenitor cells (HSPCs) that regulate self-renewal and differentiation [15]. With the rapid development of RNAi technology, the molecular understanding of gene action and regulation has been elucidated.

RNAi functions in conjunction with the target gene at the mRNA level, but RNAi also has limitations. RNAi cannot completely inhibit gene transcription and translation. It can easily become off-target, which leads to both false positives and negatives, resulting in non-target gene silencing [16]. Meanwhile, RNAi is limited to cytoplasmic screening because of the localization of the RNAi machinery in the cytoplasm [17]. It also can cause incomplete and reversible protein depletion [18]. Moreover, RNAi is not applicable to genes with low background expression levels in cells. These exogenous shRNA/siRNA/miRNAs can saturate the endogenous quantity of the cellular microRNA (miRNA) of cells, thus affecting the synthesis of endogenous miRNAs, which leads to cytotoxicity and even cell death [19].

### 2.2. Overexpression of cDNA/ORF

Gene overexpression technology refers to constructing coding sequences/open reading frames (CDS/ORFs) of target genes and transferring them into cells through a plasmid or virus vector to cause significant gene transcription and translation, thus achieving target gene overexpression (Figure 1B, Table 2). The overexpression of cDNA is used to reveal functional genomics. A studied gene may belong to a family. The family has many genes with similar structures and mutual compensation functions. Therefore, when one or two genes are knocked out, phenotypic changes are not observed; thus, studying the gene’s function is difficult. In this situation, overexpression can enhance the abundance of gene expression products. By comparing the resulting phenotype with wild-type cells, overexpression can be combined with high-throughput sequencing and used to study functional genomics.

The overexpression of cDNA is widely used to screen gene targets and study functional genomics. In genome-scale genetic screening, people screen gene targets by constructing cDNA libraries, overexpression or introducing foreign genes, and observing phenotypic changes [20,21,22]. Since overexpression can significantly increase the expression level of specific genes, it is beneficial to directly observe the corresponding cellular superficial changes after gene expression. Therefore, overexpression screening provides a powerful means for functional genomics’ identification [23,24], pathogenesis analysis [25], and cell phenotype acquisition [26]. One of the most classic applications is the identification of four human-induced pluripotent cell (hiPSC)-inducible transcription factors using lentivirus-mediated human tissue factor (TF) overexpression library screening, as was conducted in 2021 [26]. This research suggested that large-scale combinatorial screening could complement other cell engineering strategies that are based on developmental and computational systems biology. In 2022, critical regulators of T-cell proliferation were identified through overexpressed ORF libraries. These findings support several strategies for improving next-generation T-cell therapies [27].

Although gene overexpression provides a powerful method for inferring functional genomics, many challenges remain in practical application. For example, high exogenous gene expression presents a type of stimulus pressure on cells and cannot reflect the function of genes when they exceed the demands of the cells, which produces false positive or negative results. Furthermore, most genes are subject to alternative splicing and thus express many transcript isoforms. Thus, it is difficult to fully represent the transcript diversity using a cDNA library. In addition, cDNA libraries are expensive to construct, and the overexpression of cDNA cannot be used to study no-coding gene (lncRNA, miRNA, etc.) functions [28].

## 3. CRISPR Screening

The Clustered Regularly Interspaced Short Palindromic Repeat (CRISPR) system is an immune system acquired by archaea and bacteria, which helps to resist the invasion of foreign genetic materials, such as viruses and bacteriophages [29,30]. The CRISPR/Cas system consists of CRISPR sequences (including leader, conservative repeat, and spacer), transactivating crRNA (tracrRNA), protospacer adjacent motif (PAM), and genes encoding the Cas protein [30]. The Cas9 nuclease in the type II CRISPR/Cas9 system is the most commonly used system [31]. The steps of genome editing using CRISPR-Cas are: 1. Expression (and processing) of CRISPR RNA; 2. Loading of sgRNA into Cas9 nuclease; 3. Search for the PAM motif; 4. DNA unwinding and probing for sgRNA complementarity; 5. DNA cleavage; and 6. dsDNA break repair [32].

Cas9 nucleases are directed by gRNA to the target sequence to produce DNA double-strand breaks (DSBs). Two repair mechanisms are present in mammalian cells: (1) non-homologous end joining (NHEJ) and (2) homology-directed repair (HDR) [33,34].

The repair mechanism of NHEJ introduces random base insertions/deletions (INDEL), resulting in code shift mutation [35]. Dead Cas9 (dCas9) loses its cutting function after the inactivation of the two catalytic domains of Cas9, HNH (H840A mutation) and Ruvc (D10A mutation) [36]. However, it retains the ability to target and bind to DNA under the guidance of sgRNA. The CRISPR activation (CRISPRa) system and CRISPR inhibition (CRISPRi) system, based on dCas9, can fuse transcription regulators to target transcription initiation sites under gRNA guidance and activate or inhibit gene transcription. Thus, the sgRNA library based on the CRISPR gene editing system and high-throughput sequencing can be used to knock out, activate, and/or inhibit genes within the whole genome.

**Figure 2:**
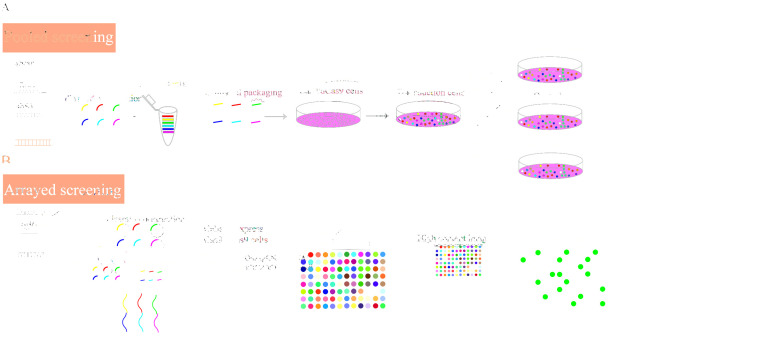
High-throughput screening mainly includes two types of screening. (**A**) The first is pooled screening, which involves the design of sgRNA/shRNA/ overexpressed cDNA libraries, constructing viral vectors, packaging of viruses, infection of target cells, drug, and/or stress screening. The aim is to determine positive screening or negative screening, and next-generation sequencing is used to detect enriched or depleted gRNA to obtain candidate genes. (**B**) In arrayed screening, the main process consists of library design and infecting or transfecting the target cells with plasmid vectors, viral vectors, and/or oligos. One gene’s sgRNA/shRNA/siRNA/cDNA is transferred into each well of a 96-well/384-well plate (different color). After screening, high-intension imaging is performed to analyze the relationship between genotype and phenotype (green). The candidate target genes are identified.

Pooled screening is a common form of CRISPR screening because of its high throughput, low cost, and simple operation, and is generally used for single known phenotypes, but some unknown valuable phenotypes may sometimes be ignored. In addition, weaker target phenotypes may be missed due to screening system efficiency. By contrast, arrayed screening uses a 96- or 384-well porous plate format. Each well contains sgRNAs specific to a target gene, which are introduced into the cells through lentiviruses, the transient transfection of plasmids, or antisense oligonucleotides. This format allows for the measuring of a diverse range of phenotypes through drug or fluorescence screening by high connotation instruments and time delay microscopes [37]. Arrayed screening can directly correlate the observed phenotypes with interference. Therefore, arrayed screening can be selected when the overall phenotype is not obvious after knock-out or interference. When compared with pool screening, growth and phenotype upon transcriptional repression are not influenced by the presence and growth of competing clones [38]. Arrayed filtering is suitable for smaller library filtering. However, arrayed screening library construction is complicated, the workload is large, and the cost is high. Recently, the construction of a human whole-genome arrayed screening library has been reported [39], which is of great significance for the systematic study of human functional genomics. At the same time, we also expect technological progress to promote the construction of other model animal whole-genome arrayed screening libraries (Figure 2).

### 3.1. CRISPR Activation Screening

**Figure 3:**
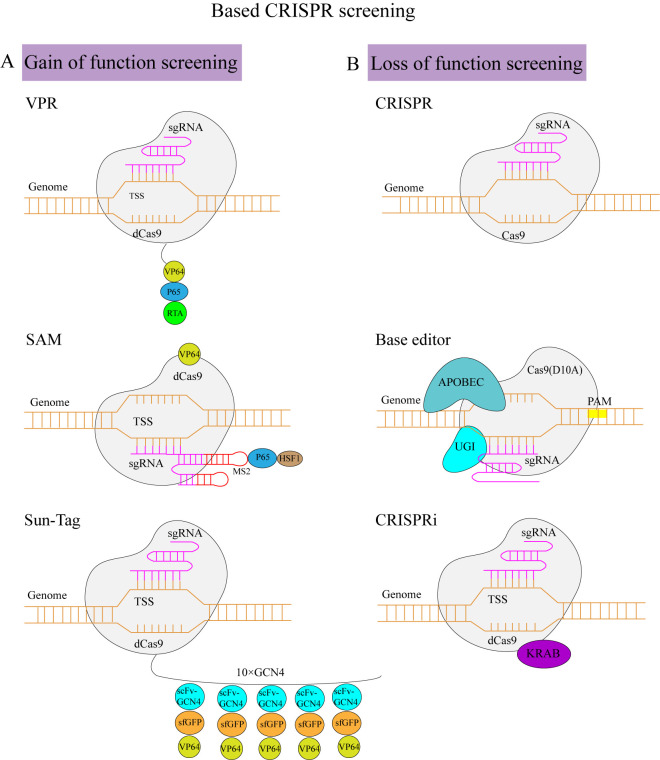
(**A**) CRISPR-based gain-of-function screen technology: (1) VPR, (2) SAM, and (3) Sun-Tag. VPR mainly recruits using the dCas9 fusion transcription activators VP64, p65, and RTA. The SAM complex recruits using three different activators, VP64, p65, and HSF1, and Sun-Tag represents the dCas9 fusion short-peptide SCFVGCN4-SFGFP-VP64. (**B**) CRISPR-based loss-of-function screen techniques: (1) CRISPRko, (2) CRISPR base editor, and (3) CRISPRi. CRISPR mainly introduces mutations using Cas9 nuclease, which cleaves double strands and causes HDR or NHEJ. The CRISPR base editor uses a deaminase to change the base of the editing window to interfere with gene expression. CRISPRi inhibits gene transcription mainly via the steric effects of dCas9 and the transcriptional suppression domain, Krüppel-associated box (KRAB).

By fusing dCas9 with the transcriptional activation element, the transcriptional activation of specific genes can be achieved through sgRNA mediation (Table 2). Gain-of-function screening can be carried out by designing the sgRNA of the target genes and then establishing the sgRNA library using lentiviruses as the carrier. Afterward, the infected cells are screened by drugs or phenotypes to obtain the target cells, which are then sequenced using high-throughput screening to detect the sgRNA-targeted genes. sgRNA recruits dCas9 into the target gene promoter region to improve gene expression. When dCas9 binds to the promoter, the transcriptional activator promotes RNA polymerase recruitment to start the transcription [40]. Generally, the highest levels of activation can be achieved by targeting within the −400 bp to −50 bp window [41,42,43]. Three major transcriptional activation systems have been identified: (1) dCas9-VPR, (2) dCas9-synergistic activation mediator (SAM), and (3) dCas9-SunTag. The first type is dCas9-VPR [44] (Table 3). The ternary transcription-activating factors of VPR, namely, VP64, p65, and R transactivator (Rta), are fused in dCas9 in the TSS region under the guidance of sgRNA. In addition to facilitating the direct fusion of the transcription-activating factor with dCas9, the transcription-activating factor can also be recruited around dCas9 via a molecular linkage. The second type is dCas9-SunTag [45]. Under the guidance of sgRNA, a Sun-Tag peptide is fused with dCas9 to recruit scFv-GCN4, sfGFP, and VP64. The third type is the dCas9 SAM system [41]. Combining the MS2 hairpin with the sgRNA and recruiting the activating helper proteins MCP, p65, and HSF1 to the dCas9-VP64 fusion protein can greatly improve the endogenous genes’ activation efficiency. In some cases, SAM shows a better activation effect. The up-regulation of genes can be more than 10,000-fold, but VPR, SAM, and Sun-Tag are in an order of magnitude in terms of gene expression multiplication [46] (Figure 3A).

CRISPR activation is widely used in screening. Numerous studies have shown that CRISPR activation screening can screen for key regulators of fate in complex cellular processes, including zygotic activation [47], cell differentiation [48], reprograming [49], and resistance to drugs. Traditional CRISPR screening uses simple phenotyping and high-throughput sequencing to identify the key genes that regulate cell fate. Recently, researchers combined CRISPRa screening with single-cell transcriptome sequencing to simultaneously identify the effects of different gene activation processes on cell fate [47,50]. Their single-cell transcriptomic profiling of CRISPRa-perturbed cells provided both system-level and molecular insights into these mechanisms.

CRISPRa can produce a level of overexpression closer to the physiological level compared to traditional overexpression cDNA technology by efficiently activating endogenous genes [51]. Thus, it can promote gene expression without the need for the additional construction of foreign expression originals and is not limited by the size of the transcripts. In addition, CRISPR can also be used to analyze the function of non-coding RNA [52]. Both gene overexpression technology and CRISPR activation technology can achieve the up-regulation of gene expression. However, due to the limitation of the vector capacity used by gene overexpression technology, gene overexpression technology can only be used to study genes of a certain length. CRISPR screening technology achieves gene overexpression by enhancing the transcription of the target gene promoter, which is not limited by the gene size. It is easier to design and obtain gRNA for large-scale screening using CRISPR.

CRISPR activation has efficiency problems as it is limited to the design of sgRNA and not all genes can be activated; however, overexpression can achieve this type of activation. Sometimes it is difficult to up-regulate the expression of certain genes with low background levels through CRISPR activation; thus, the gene is unavailable for screening.

### 3.2. CRISPR Knock-Out Screening

CRISPR/Cas9 can efficiently and specifically target specific DNA sequences through sgRNA and then produce DNA double-strand breaks (Table 2). A CRISPR knock-out screen contains several stages: (1) introduction of mutations, (2) selection of genes or design of a whole-genome sgRNA library, (3) cloning the synthesized sgRNA into lentivirus vectors, (4) lentivirus transduction, (5) infecting cells with a low multiplicity of infection (MOI), (6) screening cells according to resistance or phenotype, and (7) obtaining candidate genes by high-throughput sequencing combined with bioinformatics (Figure 3B).

CRISPR knock-out screening is a high-throughput, unbiased, and efficient method to identify gene functions in diverse biological processes and pathways. In a recent study, researchers used mouse embryonic stem cells carrying a pluripotent gene Rex1 fluorescence reporting system (Rex1-GFP) to systematically screen for key genes that maintain and exit naive states [53]. This helps to expand our understanding of the mechanisms of early fate determination in mouse embryos. In other studies, CRISPR knock-out screening also showed relatively stable and powerful gene function identification capabilities, such as tumor drug resistance gene identification [54], immune cell differentiation regulation genetic screening [55], and cell proliferation-related genes identification [56]. These reports provide an effective tool for engineering cell modification, tumor therapy, etc.

CRISPR/Cas9 can completely knock out proteins at the genome level; thus, the target protein’s function will be lost naturally. When compared with RNAi, the knock-out target can be extended to non-coding regions, including promoters and introns, in addition to a significantly increased signal-to-noise ratio [57]. Moreover, CRISPR knock-out is highly specific and its off-target rate is low. It is reported that CRISPRko screening is superior to shRNA and CRISPRi in identifying essential genes [58]. Knock-out causes cell death, while CRISPRi can enable varying degrees of knock-down, which is more conducive when studying essential functional genomics [59]. However, CRISPR/Cas9-based screening is based on DSBs in DNA, which can induce p53-mediated DNA damage and cell growth arrest in some cells [60], thus causing a change in transcriptome characteristics. Since CRISPRko screening is irreversible, CRISPR knock-out has cellular heterogeneity [61] and diploid cell heterozygote effects.

### 3.3. CRISPR Interference Screening

In some cases, CRISPRko leads to slow cell growth, stagnation, or even death; thus, we cannot use this method to study functional genomics. At the same time, RNAi inhibits genes at the mRNA level; thus, it is impossible to study non-transcriptional RNA fragments. Researchers have developed a dCas9-KRAB system based on dCas9 using the CRISPR technology, which directs dCas9 to target the promoter region, thus causing gene expression inhibition due to the spatial steric effect (Table 2). The optimal sgRNA design window is +25–+75 nt downstream of TSS [62]. Based on this, to improve transcriptional silencing, the fusion of dCas9 with the repressor domain (such as KRAB) [63] can reach up to 99% inhibition [64]. Reaching the target gene transcription starting site (TSS) region under the guidance of sgRNA can enable the inhibition of transcription initiation and silence target gene expression by forming heterochromatin (Figure 3B).

In 2019, Ruilin et al. identified the genes necessary for neuronal survival based on CRISPRi hybrid screening [65]. Their results highlighted the power of unbiased genetic screens in human-induced pluripotent cell (iPSC)-derived differentiated cell types and provide a platform for systematically investigating normal and disease neuronal states. In the same year, a similar combination of single-cell sequencing identified candidate regulators of human endoderm differentiation [66]. Combining single-cell RNA-seq with parallel CRISPR perturbations allowed researchers to comprehensively define the loss-of-function phenotype of factors in END development. Compared with CRISPR knock-out, the high efficiency and low cytotoxicity of CRISPRi make the state of cells with low gene function be more similar to normal cells, which can help us to better understand the maintenance of genes according to the cell state. In addition, CRISPRi has been widely used for the unbiased screening of lncRNA [67,68] and mitochondrial [37] functional genomics.

RNAi only acts on coding RNA, but compared with RNAi, CRISPRi can act on both coding and non-coding RNA. Its effect is reversible [69], and the action site is limited to the TSS, which reduces the miss effect to a certain extent. CRISPRi inhibition is more uniform and effective than that of Cas9 [70]. In terms of drug screening, CRISPRi may have more advantages than CRISPRko because the blocking of drugs against targets is generally incomplete. CRISPRi alleviates the toxic effect of DSBs caused by CRISPRko [62].

CRISPRi targeting bidirectional promoters may result in false positives, and bidirectional promoters are up to 10% of human genes [71]. CRISPRi activity is highly sensitive to mismatch between target DNA and sgRNA [41]. dCas9 causes toxicity at high concentrations, and low concentrations affect the targeting effect [72].

### 3.4. Base Editing Screening

In recent years, in addition to the typical CRISPRko screening, high-throughput screening based on base editing has been gradually applied. David Liu’s group discovered the first base editor in 2016 [73]. The base editor mainly consists of a deaminase, uracil DNA glycosyl inhibitor (UGI), and nCas9/dCas9. It mainly includes cytosine and adenine base editors, which can be used for specific point mutations (Table 2). At present, the main screening strategies are to destroy splicing sites or translation starting points through the base editor or to interfere with gene expression by the early introduction of stop codons [74] (Figure 3B).

In 2021, Ping Xu et al. established a whole-genome BARBEKO screening strategy combining a cytosine base editor (CBE) and iBARed sgRNAs [74]. Meanwhile, Ruth et al. used point mutations in CBE to identify high-throughput gain-of-function (GOF) and loss-of-function (LOF) variants and phenotypic relationships [75]. In another report, in the same year, Changcai et al. used single-base editing tools (ABE and CBE) based on CRSPR to screen and identify mutations affecting the function of breast cancer genes 1/2 (BRCA1/2) [76]. In 2022, m^6^A site promotion and the inhibition of the human embryonic stem cell endoderm was screened based on ABE [77]. This study provided a functional screening of m^6^A sites and paved the way for functional studies of m^6^A at the individual m^6^A site level. These results indicated that the base editor, as a novel gene editing technology, can be used for robust genetic screening. This screening tool is broadly useful to readily and scalably functionalize genetic variants. This valuable tool has improved the quality and efficiency of screening and is no longer subject to DNA cut-induced cytotoxicity. And base editing screens with efficiency correction are a powerful strategy for identifying pathogenic variants in a high-throughput manner. In recent years, base editing systems with wider editing sequences and higher accuracy have been continuously developed [78]. Therefore, we look forward to the development of more efficient and convenient base screening platforms to enable diversified high-throughput functional genomics.

Sometimes in response to CRISPRko screening based on DSBs, cellular DNA cleavage damage causes the impression of apoptosis or death. Therefore, it is difficult to screen related phenotypes. CRISPRko screening based on the base editor does not cause DSBs [79]. Thus, it is widely used in the functional screening experiments of some primary cells that are more sensitive to double-strand damage. However, some problems limit the application of base editing screening. For example, the current base editor is limited to four base conversions, and the editing window is limited; thus, the design and coverage of sgRNA have great limitations [80]. On the other hand, some base editors can generate multiple types of edits in the editing window class [81]. It is impossible to confirm which genotype determines the observed phenotype directly; thus, subsequent multiple verifications are required. We also look forward to a method that can capture the mutation type of the corresponding cell while detecting base-editing screening sgRNA. Thus, we can achieve the joint analysis of the genotype and its corresponding degeneration, which will allow us to preliminarily identify the changes in cell function caused by single base mutations and have a profound impact on the clinical diagnosis and research of rare diseases and tumors.

## 4. Screening Delivery Systems

**Figure 4:**
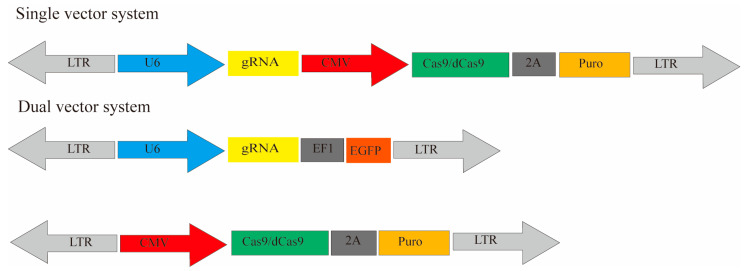
The single-vector system consists of sgRNA and Cas9/dCas9, while the dual-vector system constructs sgRNA and Cas9/dCas9.

Current genetic screening vectors mainly include plasmids, lentiviruses, adenoviruses, adeno-associated virus vector (AAV), retroviral vectors [25], and nanoparticles. According to the experimental requirements, a single- or dual-vector system can be constructed. The library and activation/interference vectors are expressed concurrently for a single-vector system. A single-vector system can be used for cDNA overexpression, RNAi, and CRISPR. For dual-vector systems, two separate vectors need to be transduced into the cells together, which is common in a CRISPR system (Figure 4). Take CRISPR as an example. The CRISPR/Cas9 library plasmid is a single-vector system, including promoters, Cas9, gRNA, and screening markers. Its advantage is transfecting Cas9/dCas9 and gRNA simultaneously. The operation is relatively simple, but the transfection efficiency may be affected since the plasmid is too large. The other is a dual-vector system. This system has the advantage of first constructing stable cell lines expressing Cas9 or dCas9 and then infecting them with gRNA libraries. This system can be applied to different libraries to facilitate the screening of cell lines with different conditions. Thus, it is more convenient to use.

Plasmid vectors are usually delivered to cells by transfection. Its advantages lie mainly in its simple technology and the high expression levels of genes carried on the vector. The disadvantage is that the transfection of the plasmid vector is transient. The vector exists in cells mainly in the form of free DNA, which is not integrated into the host genome. Meanwhile, the types of cells transfected with the plasmid are limited, and transfection efficiency varies greatly among different cell lines. In addition, the number of copies of plasmid-transfected genes in each cell is highly heterogeneous. Another limitation of plasmid transfection is that plasmids are diluted and degraded over time. In a CRISPR-Cas9/dCas9 system, plasmid vectors co-expressing Cas9 and gRNA can be found in addition to single plasmids expressing Cas9 or gRNA. In screening, high-throughput phenotypic screening can be achieved by transfecting plasmid vectors with shRNA/ overexpressed cDNA [82,83] (Table 4).

Lentivirus vectors can be effectively integrated into the target cell genome by constructing plasmids, packaging lentiviruses, and infecting cells. Its advantage is that the effect of lentiviruses on target genes is stable and permanent. Additionally, lentiviruses can be transferred to a wide variety of cells, and compared with conventional transfection, lentivirus transduction results in relatively uniform gene copy in cells. In addition, the virus produced by the lentivirus vector cannot replicate in target cells, which is of high safety. The insertion of the lentiviral vector is biased (Table 4).

Adenovirus vectors are efficient virus vectors. Their advantage is the low risk of mutation and random insertion into the genome since they do not integrate into the genome after entering the target cell. However, this means they are easily lost. In addition, adenoviruses can be used for cell transduction in vitro and in vivo. Like lentiviruses, they can only be transduced into cells but cannot replicate. They have a limited variety of transduced cells. Meanwhile, adenoviruses can induce a strong immune response to the cells in vivo, thus affecting the transduction efficiency. Adenoviral vectors are mostly used for RNAi and overexpression (Table 4).

The obvious advantage of AAVs is their low immunogenicity and being relatively safe, making them an ideal tool for in vivo cell experiments. AAVs also have replication defects; therefore, they have high security. The structure of AAVs is simple and stable. However, AAVs have a smaller insert size capacity than both lentiviruses and retroviruses. AAV delivery is limited to non-dividing cells. Additionally, the virus is packaged, and the time cycle for titer determination is long. AAVs cannot integrate DNA into the genome [84] (Table 4).

Generally, two types of transposon systems can be found: (1) Sleeping Beauty (SB) and (2) piggyBac (PB). A transposon system includes two functional components: (1) transposase enzyme and (2) terminal inverted repeats (TIRs). After targeting the cells, the transposase enzyme recognizes the long-terminal repeat (LTR) region of the transposon, and transposon elements are inserted into the target cell genome. While the accessory plasmid is gradually lost over time, the transposon becomes permanently integrated, findings that highlight the definitive advantage of transposon vectors. Moreover, transposition can be achieved through conventional transfection methods, rendering the process relatively straightforward and immune-reaction-free, although it may have limited cell applicability (Table 4).

A retrovirus integrates into the cell genome and only transduces dividing cells, and its immunogenicity is relatively low (Table 4). In 2021, Zhang Feng’s team developed a new delivery approach, the Selective Endogenous eNcapsidation (SEND) [85]. They demonstrated SEND as a modular platform suited for development as an efficient therapeutic delivery modality. Moreover, SEND, a fully endogenous system, may have reduced immunogenicity and higher safety compared to the currently available viral vectors.

Viral vectors are regarded as the most comprehensive delivery tool for CRISPR-Cas9. In addition to viral delivery systems, another approach, nanoparticles (NPs), has been successfully employed in delivering Cas9-sgRNA complexes based on the construction of large ribonucleoprotein–NP complexes, such as lipidoid nanoparticles [86,87], gold nanoparticles [88,89,90], DNA nanoclews [91], black phosphorus nanosheets (BPs) [92], graphene oxide (GO) nanosheets [93], and zeolitic imidazolate framework (ZIFs) [94]. Nanoparticles have advantages, especially their controllable size, good loading capacity, and bioresponsive behavior [95,96,97]. However, they are limited by delivery difficulties for large sizes and low editing efficiencies. One of the most well-developed methods for delivery is lipid nanoparticles (LNPs). They advantages lie in the manner of endocytosis. LNPs can electrostatically fuse with the cell membrane by inverted non-bilayer lipid phases. On the other hand, the difficult is that LNPs can be exocytosed. Recently, an increase in the number of modified nanoparticles has been reported to increase the delivery efficiency for the widespread application of gene editing, which provides hope for basic research and the clinical treatment of various diseases [98,99,100,101,102,103,104,105,106,107,108,109,110].

## 5. Bioinformatics Analysis

After infecting the cell pool with a low MOI virus and applying joint screening, each cell has sgRNA/shRNA and the corresponding cell interference. First, after screening by drugs or drug power, the cells are collected from the control and experimental groups, and the DNA is extracted. Then, the sgRNA/shRNA is amplified by PCR with universal primers to obtain the number of reads of each type of sgRNA in the sample. The abundance of sgRNA/shRNA is determined in the interfered cells to obtain the corresponding candidate genes. Some commonly used bioinformatics algorithms for analyzing genome-wide screening results are currently available. The earliest algorithms, edgeR [111], DESeq [112], baySeq [113], and NBPSeq, are not specifically designed for CRISPR screening, but they can be used to analyze gRNAs with significant differences to identify candidate genes. RNAi gene enrichment ranking (RIGER) and redundant siRNA activity (RSA) can be used to analyze the data generated by RNAi screening. RIGER identifies key genes in genome-scale shRNA screening and RSA for siRNA screening. Several algorithms are also dedicated to CRISPR/Cas9 screening: (1) HiTSelect [114], (2) MAGeck-RRA [115], (3) MAGeck-MLE [116], (4) ScreenBEAM [117], (5) STARS [118], (6) MAGeCKFlute [119], and (7) BAGEL [120]. In addition, CRISPR screening combined with single-cell RNA sequencing can directly detect different sgRNAs from a single cell. Related algorithms include MixScape [121], MIMOSCA [122], and scMAGeck [123]. For subsequent functional genomics screened by different high-throughput screening algorithms, cluster analysis [124], gene function analysis (GO) [125], route enrichment analysis (Kyoto Encyclopedia of Genes and Genomes [KEGG]) [126], and others are mainly used.

## 6. Joint Applications with Other Technologies

**Figure 5:**
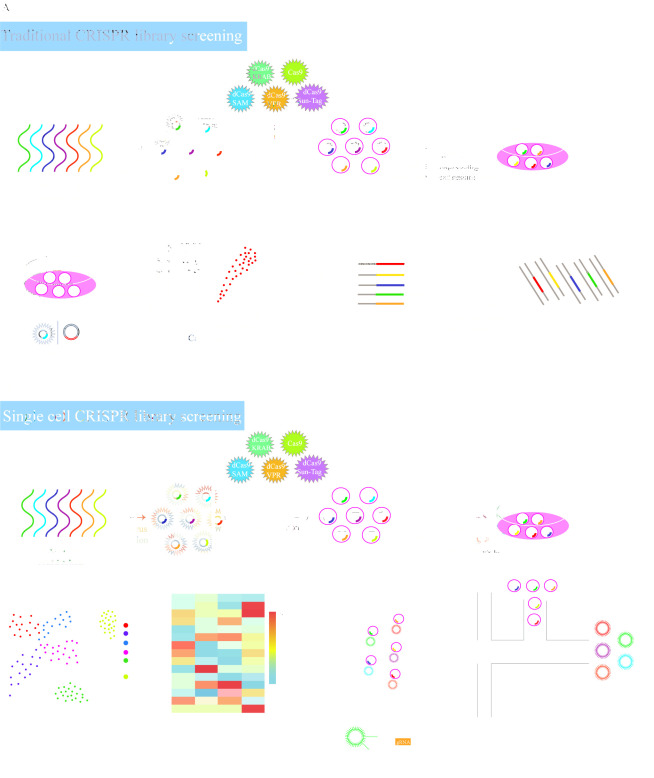
(A) Conventional screening strategy: (1) construct sgRNA library, (2) package and generate the lentivirus, (3) infect the target cells, (4) collect the cell populations after screening, (5) apply second-generation sequencing, (6) obtain enriched genes, and (7) perform functional verification. (B) Single-cell screening strategy: (1) construct sgRNA library containing cell labels, (2) infect the target cells, and (3) perform single-cell sequencing. Segments of rings with different colors represent different sgRNA unless otherwise stated.

Library screening usually uses low-dimensional phenotypic screening methods, such as cell survival and specific gene expression screening, followed by enrichment analysis and individual gene function verification [127]. These methods are used to obtain limited phenotypic information and do not identify the gene perturbations that cause cell phenotypic differences [128]. As a solution for these problems, single-cell CRISPR screening concurrently reads out genetic perturbations and changes in cellular transcriptional information in individual cells, thus closely linking the genotype to phenotype (Figure 5).

**Figure 6:**
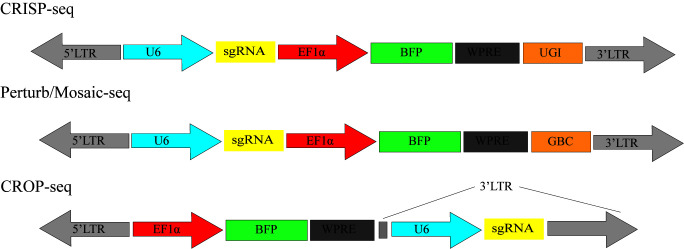
CRISPR screen is combined with single-cell sequencing to capture transcriptome information of both sgRNA and individual cells using CRISP-seq/Perturb-seq/Moscai-Seq/CROP-seq by introducing a sgRNA library with cell-specific barcodes.

In recent years, technologies such as CRISP-seq, Perturb-seq, CROP-seq, and Mosaic-seq (Figure 6) have made it possible to comprehensively analyze the relationship between gene perturbation and cell phenotype using high-throughput screening. Mosaic-seq allows the unambiguous linkage of each sgRNA to a single barcode in the plasmids. In this way, we can determine a single cell’s transcriptome information and sgRNA through single-cell RNA-seq. This method was first reported in 2017 and established the relationship between enhancers and cell gene expression [129]. Similarly, CRISP-Seq uses scalable lentivirus vectors, including gRNA and transcribed UGI. Each gRNA corresponds to one UGI. The main function of UGI is to identify gRNA from single-cell RNA seq data [130]. However, the separation of UGI and gRNA leads to the misreading of sgRNA during sequencing. In addition, accurate library construction remains a considerable challenge. In response, Perturb-seq and CROP-seq read the information by directly capturing the gRNA. However, the specific gRNA structure and library skeleton make the previous libraries not fit for general purposes, and in addition, CROP-seq is incompatible with some gRNA delivery systems [131] (Figure 6). Recently, researchers have used this approach to reveal a multidimensional portrait of cell behavior, gene function, and regulatory networks in cells’ different life activities [50,132]. The development of single-cell CRISPR screening technology advances the goal of creating a comprehensive genotype–phenotype map.

The above scRNA-seq-based methods greatly improve the scalability of library screening, but are not compatible with the complex and dynamic cell phenotypes caused by gene perturbations. In 2019, Feldman et al. developed an optical pooled screen that can systematically analyze genetic components and conduct phenotypic analysis with a wide range of spatial and temporal definitions after gene perturbation [133]. The development of an optical pool screen that can observe dynamic phenotypic changes in cells is exciting [134].

Key functional genes can be identified through library screening, and the next step is to map the changes in cell locus after key gene perturbations, thereby providing insights into gene function. Recently, CRISPR/Cas9 has been used in combination with DNA barcoding to construct the dynamic trajectories of cell division, which have made it possible to reconstruct the development of lineage relationships at a single-cell resolution [135,136,137,138,139]. In a recent study, researchers developed a lineage-tracing system called CREST (CRISPR editing-based lineage-specific tracing) by the tissue-specific induction of Cas9 expression [140]. In this way, they precisely mapped the single-cell lineages in developing mouse ventral midbrain, identified the origin of dopaminergic neurons, and demonstrated that the transcriptome defined progenitor cell types with distinct clonal fates and molecular features. In recent years, advances in lineage tracing have made it possible to map genetically disturbed cell lineages simultaneously with library screening. In addition, in situ read lineage tracing [141] combined with optical pooled screen is a development direction of CRISPR screening, which has important significance for understanding the diverse phenotypes and mechanisms of cells.

Single-cell CRISPR screening can also detect the alterations in chromatin state after CRISPR interference [142,143,144]. Meanwhile, it can also detect protein levels. CITE-Seq [145] and ECCITE-seq [121], by constructing an antibody with labeled DNA, can simultaneously detect a single cell’s transcriptome, gRNA, and protein expression. CRISPR screen combined with spatial transcriptomics constructed a protein-based vector/cell bar-coding system, the Pro-Codes, which is composed of triplet combinations of linear epitopes fused to a scaffold protein, dNGFR [146]. It can identify regulatory factors affecting tumor microenvironments.

## 7. Conclusions and Future Prospects

As the most popular tools, high-throughput genome-wide screening based on RNAi, overexpression libraries, and CRISPR has been widely used in screening essential genes for cell survival/proliferation/death, drug resistance genes, drug-sensitive genes, important target genes of signaling pathways, and/or related mechanisms.

The hidden challenges of the current high-throughput screening in applications include off-target effects. First, for a library’s design, high coverage and high targeting rates are required to accurately study the function of targeted genes. Second, gain/loss-of-function screens exhibit predilections not only in alterations of intracellular gene expression levels but also influenced by cell heterogeneity. Lineage tracing can be performed in combination with barcoded/inducible libraries or single-cell sequencing for cell heterogeneity. The third involves the selection of appropriate delivery vectors. Delivery systems are limited by the delivery efficiency and immunogenicity in some primary cells. Consequently, it is urgent to develop efficient and safe delivery systems. High-throughput screening in vivo has always been a challenge due to delivery efficiency and delivery scalability limitations. Therefore, we look forward to the development of novel delivery systems combined with spatial transcriptome screening to enable the functional identification of key fate regulators in vivo.

The applications of CRISPR screening are still limited in some cases, including multiple factors’ function screening and data integration analysis. The cell fate regulatory networks are usually governed by multiple core factors [48]. However, the current screening technology options are not adequate. For example, for the CRISPR/Cas9 screening of 100 genes, six gRNA per gene are designed to ensure the knock-out efficiency of different isomers, and the simultaneous knock-out of 2 genes produce hundreds of thousands of combinations, and the synergies of more factors are unimaginable. For multifactor functional screening, an optimal sgRNA design is important to reduce the number of sgRNA required for library screening. In addition, the development of computational algorithms based on artificial intelligence or deep learning is of great significance in helping to build the fate regulator interaction network. Technological breakthroughs and the development of sophisticated databases and tools will help researchers to expand their understanding of the synergies of different factors and advance the further development of CRISPR screening.

## Figures and Tables

**Table 1:** Three types of endogenous or exogenous RNA molecules.

Type	Size	Source	Dicer Enzyme Processing Method	Target Site	Mechanism of Action	Cellular Delivery Method
shRNA	50–100 base pairs	Local complementary paired double-stranded RNA formed by a hairpin structure	Double-stranded RNA cleavage on both strands	Arbitrary position of mRNA	mRNA degradation (transcriptional level regulation)	Requires vector, such as lentiviral transduction shRNA library
siRNA	20–25 base pairs	Artificially synthesized linear double-stranded RNA with fully complementary base pairing in the duplex region	Double-stranded RNA cleavage on both strands RNA	Arbitrary position of mRNA	mRNA degradation (transcriptional level regulation)	Transfection of dsRNA or siRNA
miRNA	21–23 nucleotides	Local duplex formed by a hairpin structure, often with partial complementarity	Stem-loop removal	Target gene 3′-UTR region	mRNA degradation (transcriptional level regulation) or inhibition of mRNA translation (translational level regulation)	Transfection of miRNA

shRNA: small hairpin RNA; siRNA: short interfering RNA; miRNA: microRNA.

**Table 2:** Functional genomics screening techniques comparison.

Functional Genomics Screening Technique	Element	Toxicity	Off-Target Effect	Gain/Loss-of-Function Type	Reversibility	sgRNA Target Region	Adaptability
RNAi	shRNA or siRNA	Non-toxicity	High: false positives and false negatives	Knock-down	Reversible	RNA	Mature RNA in the cytoplasm
CRISPRi	dCas9/sgRNA/transcriptional repressor	Non-toxicity	Low	Knock-down	Reversible	50 bp upstream and 300 bp downstream of the TSS of genomic DNA	Coding RNA and lncRNA, i.e., the entire genome
CRISPRko	Cas9/sgRNA	DSB toxicity	Low	Knock-out	Irreversible	Arbitrary target site of genomic DNA	Coding RNA and lncRNA, i.e., the entire genome
Base editor	dCas9/sgRNA/APOBEC1	Non-toxicity	Low	Base change	Irreversible	Arbitrary target site of genomic DNA	Genomic DNA
cDNA overexpression	cDNA library plasmid	Non-toxicity	-	Overexpression	Irreversible	Arbitrary target site of genomic DNA	Genomic DNA
CRISPRa	Cas9/sgRNA/transcriptional repressor	Non-toxicity	Low	Activation	Reversible	50–500 bp upstream of the TSS of genomic DNA	Coding RNA and lncRNA, i.e., the entire genome

**Table 3:** Three systems based on dCas9.

System	Transcriptional Activation Element	Molecular Tether
dCas9-VPR	VP64, p65, Rta	-
dCas9-Sun Tag	scFv-GCN4, sfGFP, VP64	Multimeric GCN4
dCas9-SAM	MCP, p65, HSF1	MS2 Hairpin

**Table 4:** Viral vector types for CRISPR screening.

Vector Type	Genome Type	Immunogenicity	Integration into Host Genome	Range of Infected Cells	Uniformity of Copy Number Post-Transfection/Transduction	Security and Reasons	Persistence of Exogenous Gene Expression	Packaging System
Plasmid vector	dsDNA	None	Non-integrating	Limited; difficult to transfect neurons and other cells	Non-uniform	Secure	Transient expression	Plasmid
Lentivirus vector	RNA	Low	Random integration	Broad; nearly all mammalian cells	Uniform	Secure 1. Viral packaging requires helper plasmid. 2. 5′ LTR inactivation.	Stable	One transfer plasmid, one membrane protein expression plasmid, two packaging plasmids
Adenoviral vector	dsDNA	High	Non-integrating	Relatively widespread; however, difficult to transduce endothelial cells, neurons, etc.	Uniform	Secure 1. Can transduce target cells only. unable to replicate. 2. Viral packaging requires helper plasmid.	Transient expression	Transfer plasmid containing adenoviral genome sequence with the deletion of E1/E3 genes
AAV vector	ssDNA	Extremely low	Non-integrating	Relatively widespread; different serotypes correspond to different types of cells	Uniform	Safest, with replication defects	Transient expression	Transfer plasmid, Rep and Cap expression plasmids, and one helper plasmid
Transposon vector	dsDNA	None	Integrating	Limited; difficult to transfect neurons and other cells	Non-uniform	Secure	Stable	Helper plasmid encoding transposase, transposon plasmid
Retroviral vector	RNA	Low	Random integration	Limited; cannot infect non-dividing cells	Uniform	Secure	Stable	Transfer plasmid, membrane protein expression plasmid, packaging plasmid
